# Cumulative dissipated energy in eyes with and without corneal opacity

**DOI:** 10.22336/rjo.2022.45

**Published:** 2022

**Authors:** Cagri Ilhan, Ayse Gul Kocak Altintas

**Affiliations:** * Hatay Mustafa Kemal University, Tayfur Ata Sokmen Medicine Faculty, Hatay, Turkey; ** University of Health Sciences, Ankara Ulucanlar Eye Education and Research Hospital, Ankara, Turkey

**Keywords:** cataract, CDE, corneal opacity, cumulative dissipated energy, phacoemulsification

## Abstract

**Objective:** To compare ultrasonic energy delivered into the eye [cumulative dissipated energy, (CDE)] and frequencies of required auxiliary surgical methods during phacoemulsification surgery in eyes with and without corneal opacity.

**Methods:** The study was designed as a retrospective comparative observational study. The study group [Corneal Opacity Group, (COG)] was comprised of 31 eyes of 31 cataract patients with corneal opacity. Only nebular and macular corneal opacities (according to slit-lamp based classification of Agrawal) were included in the study. The control group (CG) was comprised of 40 eyes of 40 cataract patients without corneal opacity. The CDE values were obtained using the Centurion system (Alcon, Fort Worth, TX) and the patients were followed-up postoperatively for a period of one month.

**Results:** The mean age of the subjects was 71.46 ± 8.86 years (52-89) in COG and 66.12 ± 5.96 years (55-80) in CG (p >0.05). In COG, the most common etiologic factors were trauma, keratitis, and degenerative diseases. The mean CDE value was 15.16 ± 8.71 (2.20-42.65) in COG and 10.04 ± 6.28 (3.77-31.80) in CG and it was found as significantly higher in COG (p=0.003). Some auxiliary surgical methods including posterior synechiolysis and anterior capsule staining were more commonly performed in COG (p=0.044 and p=0.040, respectively). No intraoperative or postoperative complication was observed.

**Conclusion:** More ultrasonic energy is delivered into the eye and more auxiliary surgical methods are needed in cataract patients with corneal opacity who underwent phacoemulsification.

**Abbreviations:** CDE = Cumulative dissipated energy, COG = Corneal Opacity Group, CG = Control group, IOL = Intraocular lens, LOCS = Lens Opacities Classification System, BCVA = best-corrected visual acuity, SRK/T = Sanders, Retzlaff, and Kraff theoretical, OVD = ophthalmic viscosurgical device, SPSS = Statistical Package for the Social Sciences

## Introduction

Cataract and corneal opacity are the most important causes for visual loss and blindness worldwide. A clear media is essential for cataract surgery and the triple procedure, including simultaneously performed cataract extraction, intraocular lens (IOL) implantation, and penetrating keratoplasty is a surgical option for cataract patients with corneal opacity [**1**]. This procedure cannot be considered a standard approach for all patients due to the risk of inaccuracy in IOL power calculation, inadequate cortical cleaning, and expulsive haemorrhage [**2**]. Additionally, this procedure carries some keratoplasty associated problems including long waiting time for suitable cornea, astigmatism induction, expulsive choroidal haemorrhage, or graft failure [**3**,**4**]. Phacoemulsification and IOL implantation may be better options for some cataract patients with corneal opacity depending on localization, size, and severity of the opacity [**5**,**6**].

The phacoemulsification system is based on cataractous lens emulsification with ultrasonic energy transformed by electric energy. The Centurion system (Alcon, Fort Worth, TX) gives an opportunity to monitor ultrasonic energy delivered into the eye as cumulative dissipated energy (CDE). Cataract patients with corneal opacity are considered challenging cases, and some auxiliary methods or surgical manipulations and ultrasonic energy out of standard can be required during surgery. The purpose of this study was to compare ultrasonic energy delivered into the eye, CDE values, and frequencies of required auxiliary surgical methods during phacoemulsification and IOL implantation procedure, between eyes with and without corneal opacity.

## Methods

This retrospective comparative observational study was carried out in a tertiary referral centre in Ankara, Turkey. The study was performed per the Declaration of Helsinki for human subjects and the study protocol was approved by the Local Research Ethics Committee. Written consent was obtained from each subject before the surgical procedure.

Medical records of the cataract patients who underwent surgical intervention were retrospectively investigated. The study group [Corneal Opacity Group, (COG)] was comprised of patients with the following inclusion criteria: 1) ≥ 18 years old; 2) grade 3 or 4 nuclear sclerotic cataract according to the Lens Opacities Classification System (LOCS) III [**7**] (if corneal opacity did not allow the determination of the classification of cataracts, it was confirmed by using dense cataract settings in the phacoemulsification platform); 3) nebular (superficial stromal scar, allowed detailed iris visibility) or macular (depth of scar up to half of stroma, obscured iris details, and allowed pupillary margin visibility) type corneal opacities according to slit-lamp based classification of Agrawal [**8**]. Patients were excluded if they had leukoma (depth of scar more than half of stroma, obscured iris and pupil details completely) and adherent leukoma (resulted from healing of perforated corneal ulcer) type corneal opacities [**8**], and if they had received combined procedurals with keratoplasty. Patients were also excluded if they had a diagnosis or clinical signs of congenital corneal opacities or developmental eye abnormalities (e.g., Peter’s anomaly, microcornea, macrocornea, microphthalmia, macrophthalmia, iris coloboma, lens coloboma, or microspherophakia) and history of congenital glaucoma. The aetiology of corneal opacities was classified as trauma (e.g., extracted foreign body, lamellar laceration, or full thickness laceration), keratitis (e.g., herpetic keratitis, bacterial keratitis, or corneal ulcer), degenerative diseases (e.g., corneal opacity after received pterygium surgery, or Terrien’s marginal degeneration), inflammatory diseases (e.g., chemical injury), and ectatic diseases (e.g., keratoconus). The control group (CG) was comprised of randomly chosen grade 3-4 [**7**] senile cataract patients who underwent phacoemulsification and IOL implantation.

The patients who underwent a complete ophthalmological evaluation and medical histories were obtained. The best-corrected visual acuity (BCVA) was determined by a Snellen chart and the values were converted to LogMAR. Intraocular pressure values were measured with a pneumotonometer. Anterior and posterior segments (if the media allowed the visualization) were examined with a slit-lamp biomicroscope (SL-D701; Topcon, IJssel, the Netherlands). B-mode ultrasonography (Compact Touch; Quantel Medical, Cournon-d'Auvergne, France) was performed for patients with severe media opacity. Biometric values needed to calculate IOL power were obtained using LenStar LS 900 optical biometry (Haag Streit Diagnostics, Köniz, Switzerland). In patients with severe media opacity, axial length was measured with an A-mode ultrasonography. IOL power was calculated using the Sanders, Retzlaff, and Kraff theoretical (SRK/ T) formulae.

All cataract surgeries were performed under local anaesthesia by the same highly experienced surgeon (Dr. Ayse Gul Kocak Altintas) using an OPMI LUMERA 700 operating microscope (Carl Zeiss Meditec AG, Jena, Germany). Pupillary dilation was achieved with preoperative topical tropicamide 1% (Bilim Ilac San. Tic. A.S., Istanbul, Turkey) and intraoperative 0.2 mL preservative free epinephrine 1:10,000 (Galen Ilac San. Tic. A.S., Istanbul, Turkey). A clear corneal incision was made with a 2.80 mm surgical blade at the steepest axis if it could be determined; at 11-12 o’clock if it could not be determined; or on another suitable region for eyes with corneal pannus, scar, or marginal degeneration. Corneal endothelium was preserved with dispersive ophthalmic viscosurgical device (OVD), and anterior chamber and bag volume were expanded with cohesive OVD. Posterior synechiolysis or pupillary membrane peeling were performed if necessary. Iris hook was used if required pupillary dilatation could not obtained. Anterior capsule was stained with trypan blue if the retinal light reflex was not clear enough. Using retro-illumination mode of the operating microscope, a minimum 5.50 mm-diameter continuous curvilinear capsulorhexis was made. The Centurion system on torsional mode and balanced phaco tip was used, and the divide and conqueror technique was used to fracture the cataractous nucleus. A capsular tension ring (Liberty; Appasamy Saglik Urunleri, Ankara, Turkey) was implanted if zonular instability was present. After clearing of cataract material, a foldable hydrophilic acrylic IOL (Acrysof IQ monofocal IOL; Alcon, Fort Worth, TX) was implanted into the capsular bag. Corneal incisions were closed with stromal hydration and corneal suturing was performed if necessary. At the end of the surgery, the CDE value was taken from the system. A standard postoperative topical medication including moxifloxacin (Vigamox; Alcon, Fort Worth, TX) and dexamethasone (Maxidex; Alcon, Fort Worth, TX) was prescribed, and the patients were followed up for a duration of at least one month.

The Statistical Package for the Social Sciences (SPSS) 23.0 (IBM Corp., Armonk, NY) was used to analyze the study data. Descriptive data were given as mean ± standard deviations (minimum-maximum values). Comparison of the categorical values was done by the chi-square test. The Kolmogorov-Smirnov test was used to check the normal distribution of the variables. The Wilcoxon and Mann-Whitney U test were used to compare the dependent and independent variables. Statistical significance level was set as p<0.05 for two-tailed tests.

## Results

COG comprised 31 eyes of 31 patients with corneal opacity, and CG comprised 40 eyes of 40 patients with clear cornea. The mean age of the subjects was 71.46 ± 8.86 years (52-89) in COG and 66.12 ± 5.96 years (55-80) in CG. The male-to-female ratio was 17/ 14 in COG and 18/ 22 in CG. There was no significant difference between the groups as demographic features included the mean age and male-to-female ratio (p>0.05, for both).

In COG, the most common etiologic factors were trauma, keratitis, and degenerative diseases. Five patients in COG (16.12%) had previously received open globe injury repair and one patient in COG (3.22%) was functionally one-eyed. The frequencies of pseudoexfoliation syndrome, glaucoma, high myopia, and systemic co-morbidities were not different between the groups (p>0.05, for all). **Table 1** highlights the details of the clinical characteristics of the groups. 

**Table 1:** The clinical characteristics of the groups

		COG	CG	p value
Corneal opacity	Trauma [n (%)]	9 (29.03)		
	Keratitis [n (%)]	7 (22.58)		
	Degenerative diseases [n (%)]	6 (19.35)		
	Unknown etiology [n (%)]	5 (16.12)		
	Inflammatory diseases [n (%)]	2 (6.45)		
	Corneal dystrophy [n (%)]	1 (3.22)		
	Ectatic disorders [n (%)]	1 (3.22)		
Additional ocular co-morbidities	Received open globe injury repair [n (%)]	5 (16.12)	0 (0.00)	0.008
	Pseudoexfoliation syndrome [n (%)]	1 (3.22)	2 (5.00)	0.712
	Glaucoma [n (%)]	3 (9.67)	1 (2.50)	0.193
	High myopia [n (%)]	0 (0.00)	1 (2.50)	0.375
	Functional one-eyed [n (%)]	1 (3.22)	0 (0.00)	0.252
Systemic co-morbidities	Diabetes mellitus [n (%)]	6 (19.35)	9 (22.50)	0.747
	Hypertension [n (%)]	6 (19.35)	8 (20.00)	0.943
	Coronary artery diseases [n (%)]	3 (9.67)	3 (7.50)	0.743
**COG** = corneal opacity group; **CG** = control group				

**Fig. 1:**
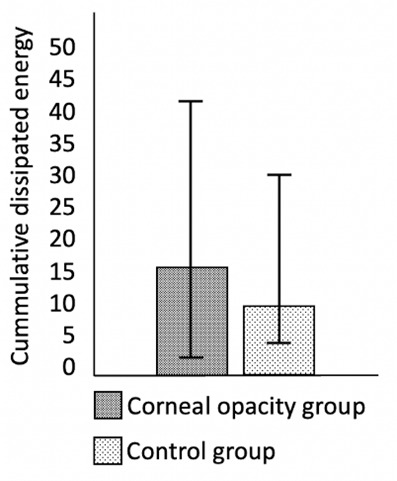
The demonstration of cumulative dissipated energy values of the groups

The mean CDE value was 15.16 ± 8.71 (2.20-42.65) in COG and 10.04 ± 6.28 (3.77-31.80) in CG. The mean CDE value of COG was found to be significantly higher than the value of CG (p=0.003). **Fig. 1** demonstrates the difference between the mean CDE values of the groups. Some surgical manipulations, including posterior synechiolysis, pupillary membrane peeling, iris hook using, anterior capsule staining, capsular tension ring implantation, and corneal suturing were more commonly performed in COG. The difference in frequencies of surgical manipulations used was statistically significant for posterior synechiolysis and anterior capsule staining (p=0.044 and p=0.040, respectively). **Table 2** emphasizes the frequencies of surgical manipulations used in the groups. No intraoperative complication was observed and IOL was implanted in the capsular bag in all patients. 

**Table 2:** The frequencies of surgical manipulations used in the groups.

	COG	CG	p value
Posterior synechiolysis [n (%)]	3 (9.67)	0 (0.00)	0.044
Pupillary membrane peeling [n (%)]	1 (3.22)	0 (0.00)	0.252
Iris hook using [n (%)]	2 (6.45)	0 (0.00)	0.103
Anterior capsule staining [n (%)]	5 (16.12)	1 (2.50)	0.040
Capsular tension ring implantation [n (%)]	2 (6.45)	0 (0.00)	0.103
Corneal suturing [n (%)]	1 (3.22)	0 (0.00)	0.252
**COG** = corneal opacity group; **CG** = control group			

At the baseline, the mean BCVA was 1.34 ± 0.41 logMAR (2.00-0.30) in COG and 0.56 ± 0.20 logMAR (2.00-0.30) in CG. On the 1st month postoperative follow-up visit, the mean BCVA was 0.19 ± 0.26 logMAR (1.00-0.00) in COG and 0.08 ± 0.11 logMAR (0.30-0.00) in CG. The mean BCVA of COG was significantly lower than CG at the baseline and on 1st month postoperative visit (p<0.001, for both). The improvement in the mean BCVA achieved by surgery was statistically significant in both groups (p<0.001, for both). On the 1st month postoperative visit, no postoperative complication, including permanent corneal oedema, decompensation, or additional opacity, permanent loss in pupillary function, or IOL dislocation was observed in any patient.

## Discussion

Ultrasonic energy delivered into the eye during phacoemulsification surgery is presented as CDE in the Centurion system. CDE represents the efficacy of the phacoemulsification procedure, and the delivery of minimum ultrasonic energy is desirable [**9**]. According to the results of this study, required CDE is higher in eyes with corneal opacity when compared with eyes with clear cornea. This result can be explained in two ways: 1) Cataracts in eyes with corneal opacity can be denser than eyes with clear cornea. It is difficult to determine the predominant factor for visual loss or to predict visual prognosis after surgical intervention in cataract patients with moderate or severe visual impairment. This condition can be a reason for hesitation of the surgeon and for the patient to take the decision to undergo cataract surgery; late presentation for surgery is not uncommon. Additionally, trauma and intraocular inflammation, which have well-known associations with corneal opacity, can interfere with cataract progression. These conditions explain why patients with corneal opacity present with denser cataracts. Some characteristics of COG in this study support this hypothesis [having older age (even if not statistically significant), more requirement of anterior capsule staining, and having worse mean BCVA value (even if corneal opacity is another important contributor for worse BCVA)]. However, this study does not directly investigate the cataract density of the groups, and this can be a purpose for a further study. Whatever the reason, more ultrasonic energy is needed to emulsify denser cataracts [**10**]. 2) In phacoemulsification surgery, effective chopping and full occlusion of phaco tip reduce the required time and ultrasonic energy [**11**-**13**]. Effective chopping and full occlusion are provided through a “second hand”, and it needs a wide, clear zone for proper using of the “second hand”. Reduced visualization cannot benefit enough from the “second hand” and more ultrasonic energy is needed to make up this handicap. 

On torsional mode, frequency of movements in phaco tip is 32 kHz and this is 80% of the frequency of movements delivered with conventional mode. In addition, torsional mode provides nucleus emulsification with rotary oscillations, while conventional mode provides only forward and backward oscillations of the phaco tip [**9**]. Fakhry et al. [**14**] reported torsional mode, which provides lower ultrasound energy and more effective phacoemulsification when compared with the conventional mode. In this study, all phacoemulsification surgeries were performed on torsional mode. Probably, if conventional mode was used, CDE values would have been higher and the difference between the groups would have been greater. Preferred phaco tip is another important factor to affect the CDE value. Demircan et al. [**15**] demonstrated that CDE was lower with the balanced tip compared with the Kelman tip. Tjia et al. [**16**] also reported that balanced phaco tip has been specifically designed for torsional mode phacoemulsification. Balanced tip increases ultrasonic effect and reduces shaft action when compared to Kelman mini-tips. Preferred nucleofracture technique also has effects on CDE value. Davison et al. [**13**] demonstrated that lower CDE is monitored with the divide and conqueror technique when compared with the phaco chop technique. They also reported that the divide and conqueror technique is associated with lower manipulation requirements in the anterior chamber and lower risk of tissue damage [**13**]. This technique is a smart option when considering the limitation in the use of the “second hand”. In this study, all surgeries were performed using balanced phaco tip and divide and conqueror nucleofracture technique, as standard approaches. The most important aspect of this study was to demonstrate the presence of a significant difference in CDE between the eyes with and without corneal opacity, even with the use of some CDE lowering adjustments.

Lower CDE represents lower ultrasonic energy use during phacoemulsification, and it is considered a lower risk of trauma in anterior chamber structures and corneal endothelium [**14**]. Corneal endothelial cell loss is associated with clinically detectable corneal oedema, which can be permanent, and results from heat produced by phaco tip, and free radicals and fluid turbulence produced during ultrasonic energy delivering [**17**]. To monitor CDE and to know the effects of different conditions on CDE is important to prevent potential ultrasonic energy associated corneal complications in phacoemulsification surgery. According to the results of this study, although more ultrasonic energy was required in cataract patients with corneal opacity, no postoperative corneal complication including permanent corneal oedema, decompensation, or additional opacity were observed in any of the patients on 1st month postoperative visit. It seems like higher values of CDE in patients with corneal opacity do not have any clinically detectable negative effect on corneal tissue. The use of dispersive OVD, which is used in all surgeries as standard, was probably an important factor to prevent phacoemulsification associated corneal complications [**18**]. Besides, slit-lamp based clinical evaluation is not enough to make a definite conclusion, and further studies using novel imaging methods, including specular microscopy, corneal tomography, and anterior segment optical coherence tomography are needed.

Cataract surgery is considered challenging in eyes with corneal opacity due to poor visualization especially during capsulorhexis and nucleus emulsification [**19**]. Some modified surgical methods were described in cases with dense and broad corneal opacity. Surgical dyes like indocyanine green or trypan blue may assist the visualization of the anterior lens capsule [**20**]. Transcorneal, intracameral, or intravitreal illumination assisted cataract surgery have been shown to overcome most of the problems related with the scattering illumination by surgical microscope [**19**-**23**]. The surgical method was determined considering several conditions including: clinical characteristics of corneal opacities like location, size, and density, status of fellow eye, and the surgeon’s experience. Sharma et al. [**24**] reported that enough visibility may be provided during each step of phacoemulsification in eyes with a peripheral or paracentral opacity, and with at least one half of clear cornea. In this study, only nebular and macular type corneal opacities underwent surgical intervention. None of these modified surgical methods were performed as standard, and anterior capsule staining with trypan blue was more commonly performed in cataract patients with corneal opacity when compared with patients with clear cornea. In this study, corneal opacity could not be considered the only reason for anterior capsule staining, and the possibility of encountering denser cataracts in eyes with corneal opacity was another important reason. Another important finding of this study was to show that posterior synechiolysis was more commonly performed in eyes with corneal opacity. Probably, a requirement of posterior synechiolysis in eyes with corneal opacity is not directly associated with corneal opacity itself, and it is associated with previous trauma and intraocular inflammation. Although not statistically significant, some other auxiliary methods, including pupillary membrane peeling, iris hook using, capsular tension ring implantation, and corneal suturing, were more commonly performed in eyes with corneal opacity. Kocak Altintas and Ilhan [**25**] demonstrated that the requirement of these auxiliary methods in phacoemulsification surgery could increase in the presence of cataract surgery associating additional ocular co-morbidities. 

## Conclusion

This study clearly demonstrated that cataract surgery in eyes with corneal opacity cannot be considered a standard surgery, and the use some auxiliary methods could be necessary. Using these auxiliary methods, IOL could be implanted in the capsular bag in all eyes with corneal opacity, and the anatomical success of the surgery are comparable with other uneventful phacoemulsification surgeries. No permanent loss in pupillary function or IOL dislocation was observed in any patient at the end of the one-month follow-up period.

This study had some important limitations, such as being a retrospective, single-centered, small sample sized, and short follow-up study. Although a standard classification was used to include the eyes with corneal opacity, location and size of the corneal opacities were not taken into consideration. Moreover, the study primarily focused on the difference in ultrasonic energy delivered into the eye, CDE, between the eyes with and without corneal opacity. The clinical relevance of the findings was not clear enough and the eyes only underwent a slit-lamp based evaluation. Trauma was the most common aetiological factor in this study and the determination of the degree of the effects of traumatic aetiology on the outcomes was not clear enough. As far as we know, no report in literature compares CDE values of eyes with and without corneal opacity. In conclusion, more ultrasonic energy was delivered into the eye and more auxiliary methods and manipulations were needed in cataract patients with corneal opacity who underwent phacoemulsification and IOL implantation procedure.

**Conflict of Interest statement**

The authors state no conflict of interest.

**Informed Consent and Human and Animal Rights statement**

Informed consent has been obtained from all individuals included in this study.

**Authorization for the use of human subjects**

Ethical approval: The research related to human use complies with all the relevant national regulations, institutional policies, is in accordance with the tenets of the Helsinki Declaration, and has been approved by the Local Research Ethics Committee of the University of Health Sciences, Ankara Numune Education and Research Hospital, Ankara, Turkey.

**Acknowledgements**

None.

**Sources of Funding**

None.

**Disclosures**

None.
